# Mortality Analysis in Geriatric Patients With Acute Kidney Injury Admitted in the Intensive Care Unit: A Single-Center Cross-Sectional Study

**DOI:** 10.7759/cureus.54509

**Published:** 2024-02-20

**Authors:** Girish V Kumthekar, Veena Purandare, Manasi Nagarkar, Shruti Paramshetti

**Affiliations:** 1 Nephrology, Symbiosis Medical College for Women, Symbiosis University Hospital and Research Centre, Symbiosis International University, Pune, IND; 2 Internal Medicine, Symbiosis Medical College for Women, Symbiosis University Hospital and Research Centre, Symbiosis International University, Pune, IND; 3 Internal Medicine, Bharati Vidyapeeth Medical College and Hospital, Sangli, IND

**Keywords:** sepsis associated aki, geriatric aki, obstructive nephropathy, drug-induced aki, acute kidney injury

## Abstract

Introduction

Acute kidney injury (AKI) is an abrupt reduction in kidney function that causes nitrogenous waste and other waste products to be retained.

Methods

This cross-sectional study was conducted from February 2015 to January 2016. The study received approval from the Independent Ethics Committee, which included patients over 60 with AKI. The study duration was 12 consecutive months to ascertain the etiology, severity, and hospital outcomes of AKI.

Results

The common etiologies of AKI included drug-induced (25%), age-related (21.67%), cardiac (13.33%), respiratory (20%), tropical (15%), and pancreatitis (15%) cases. Another predominant etiology observed was obstructive nephropathy (55%), with the highest (37.5%) mortality rate. The distribution of patients based on KDIGO criteria showed no significant difference in mortality percentages among classes (p=0.177). Conservative management without renal replacement therapy was the most common approach to treat AKI, with a 39% mortality rate.

Conclusion

Among different causes of AKI in the geriatric age group, drug-induced AKI, and obstructive nephropathy were predominantly associated with hospital mortality.

## Introduction

Acute kidney injury (AKI) is an abrupt decline in kidney function that results in the accumulation of metabolic waste. AKI is a syndromic diagnosis with different etiologies. Acute renal injury is a prevalent condition among the elderly. Its prevalence ranges from 6.8% to 36% [1,2].

As evident in the literature, the increase in life expectancy translates into a continuous growth of the aging population [3]. In actuality, people with age more than or equal to 60 years (geriatric age group) is a population group where the prevalence of AKI has been rising at the fastest rate [2].

The factors responsible increased risk of AKI in the elderly would include but are not limited to sepsis, use of nephrotoxic drugs, dehydration, and urinary obstruction. AKI might be caused or exaggerated because of comorbidities that worsen with age such as diabetes mellitus, hypertension, immunosuppression, congestive cardiac failure, and senile nephrosclerosis (the kidney changes occurring with advanced age) [4-6]. Persistent AKI was associated with 90-day mortality in a recent study by Li et al. [7].

For elderly people with AKI, the in-hospital death rate varies from 15% to 40% [8,9]. Elderly people are more susceptible to AKI, hence identifying this high-risk group is a common step in prevention attempts. AKI preventive measures could include avoiding the use of nephrotoxic medications, optimal volume expansion, and using off-pump coronary artery bypass surgery [10,11].

AKI is a significant noncommunicable disease [12]. The predominant etiologies of AKI in a geriatric population are less well-defined [13]. We also lack specific guidelines for the diagnosis and management of AKI in the geriatric population. The present study was aimed at assessing the common etiologies and outcomes of AKI with a focus on in-hospital mortality.

## Materials and methods

This was a cross-sectional study conducted between February 2015 and January 2016. The Institutional Ethics Committee (IEC) of a tertiary care teaching hospital in western Maharashtra approved the study. A geriatric patient (age more than 60 years) with AKI who fulfilled the KDIGO criteria for the diagnosis of AKI was included. All geriatric patients diagnosed with AKI during the 12 months of the study period admitted in either medical or surgical ICUs were studied. The patients with end-stage renal disease, or those who died within 24 hours of admission, and/or who did not want to participate in the study, were excluded. We could capture the disease history of each patient enrolled. We found diabetes mellitus, hypertension, chronic kidney disease (CKD), and coronary artery disease (CAD) as predominantly present morbidities. We also looked into the dialysis requirement of patients during the hospital stay and dependency on dialysis at hospital discharge. As this was an exercise at building a hypothesis, our core are was to identify if age is a risk factor for morbidity in this subset of patients diagnosed with AKI.

KDIGO defines AKI as any of the following: increase in serum creatinine by 0.3 mg/dL or more within 48 hours or increase in serum creatinine to 1.5 times baseline or more within the last seven days or urine output less than 0.5 mL/kg/h for six hours.

Three stages of AKI are defined by KDIGO criteria as per increasing severity: Stage I: Increase in serum creatinine ≥ 0.3 mg/dL (in 48 hours) or 1.5-1.9 multiplied by baseline (in seven days); Stage II: 2.0-2.9 multiplied by baseline serum creatinine; and Stage III: 3.0 or more multiplied by baseline, increase in serum creatinine ≥ 4.0 mg/dL, or beginning of renal replacement therapy regardless of a previous KDIGO stage. The KDIGO classification is special in that it offers three stages of acute renal injury severity based on variations from the baseline for serum creatinine and drop in urine output.

We enrolled 100 patients admitted in medical and surgical ICUs in the geriatric age group fulfilling the diagnostic criteria for AKI as per KDIGO guidelines 2012. The common etiologies of AKI were found to be different in different ICUs. Hence, we acquired data on the common etiologies of AKI and tried to find out if a particular etiology had a significant association with AKI.

Statistical analysis

A Microsoft® Excel worksheet (version 2019) was used to record the data, and Statistical Product and Service Solutions (SPSS, version 28; IBM SPSS Statistics for Windows, Armonk, NY) was used for statistical analysis. A chi-square test was performed to compare the frequency and percentages of categorical data. Statistical significance was p<0.05.

## Results

Table 1 shows baseline characteristics of the distribution of patients in different age groups, gender, and location in different ICUs. We observed that 65% of patients belonged to the age group of 60-69 years, and the remaining 35% belonged to the age group of 70 years or more. Moreover, we observed a male-to-female ratio of 2.33:1. Most of the patients in this study were admitted to the medical ICU (60%), and the remaining were admitted to the surgical ICU.

**Table 1:** Baseline characteristics

	Frequency (n=100)	Percentage (%)
Age (years)		
60-69	65	65%
≥70	35	35%
Gender		
Male	70	70%
Female	30	30%
Location		
Medical ICU	60	60%
Surgical ICU	40	40%

Etiology

In this study, we found an overall mortality rate of 40%, with 25 patients from the medical ICU and 15 patients from the surgical ICU. The commonest cause of death was drug-induced AKI (n=9), followed by sepsis secondary to skin and soft tissue infection (cellulitis) (n=8) (Table 2). Table 2 shows the common etiology of AKI in the medical and surgical ICUs. In medical ICUs, drug-induced cause was the commonest etiology, followed by cardiac causes (13.33%), respiratory causes (20%), tropical causes (15%), and pancreatitis (5%). The overall mortality due to AKI in medical ICUs was 41.67%. Similarly, in surgical ICUs, obstructive uropathy (55%) was the most common cause of AKI, followed by cellulitis (25%), surgical procedures (12.5%), and intestinal obstruction (7.5%). The mortality due to AKI in surgical ICUs was 37.5%. We could not identify age as the sole etiology for high mortality in this subset of patients. 

**Table 2:** Common etiologies of AKI in medical and surgical ICUs

	Frequency	Percentage (%)	Expired	Mortality (%)
Medical ICU				
Age	13	21.67%	3	23.08%
Cardiac failure	8	13.33%	2	25%
Respiratory infections	12	20.00%	6	50%
Tropical causes	9	15.00%	3	33.3%
Drug-induced	15	25.00%	9	60%
Pancreatitis	3	5.00%	2	66.7%
Total	60	100%	25	41.67%
Surgical ICU				
Obstructive uropathy	22	55%	3	13.6
Cellulitis	10	25	8	80
Surgical procedure	3	7.5	1	33.3
Intestinal obstruction	5	12.5	3	60
Total	40	100%	15	37.5

KDIGO classification of patients at admission and mortality

Table 3 shows patient distribution according to various stages of AKI (KDIGO criteria). There was no significant difference in the percentage of mortality among different stages of AKI as per KDIGO criteria (p=0.177).

**Table 3:** KDIGO classification of patients at admission and mortality

AKI stages	No. of patients admitted	Expired	Mortality (%)
Stage I	17	5	29.41%
Stage II	38	13	34.21%
Stage III	45	12	48.89%

Coexistent illness and prognosis

Table 4 shows the association between comorbid conditions (diabetes mellitus, hypertension, CAD, and chronic obstructive airway disease) and mortality. Mortality is higher when a patient has one or more comorbid conditions (p=0.027), which is highly significant.

**Table 4:** Coexistent illness and mortality in geriatric AKI

	Total	Expired	Mortality (%)
Nil	27	6	22.22%
One or more	73	34	46.58%

Table 5 illustrates the distribution of patients across three stages of AKI. We divided the patients into the predominant modality of treatment they received. We found that 81.8% of patients in stage III required hemodialysis, while 18.2% of patients in AKI stage II required hemodialysis. Out of the total number of study participants, three patients required peritoneal dialysis. All of the patients requiring peritoneal dialysis belonged to AKI stage II. 

**Table 5:** Prognosis according to the stage of the AKI and management approach

	Conservative + surgical		Peritoneal dialysis		HD & HD + surgical	
	N	%	N	%	N	%
Stage I	17	26.6%	0	0%	0	0%
Stage II	29	45.3%	3	100%	6	18.2%
Stage III	18	28.1%	0	0%	27	81.8%
Total	64	100%	3	100%	33	100%

In our study, among 64 patients who received conservative and surgical treatment, 28.1% were in stage III, while 45.3% were in stage II. All the patients who received peritoneal dialysis were in stage II. Among the 33 patients, including HD and HD + Surgical, 18.2% of patients were stage II, while 81.8% of patients were stage III.

Follow-up

Table 6 shows the follow-up of patients who were categorized according to KDIGO criteria at the time of admission. The patients in stage III AKI were more likely to be dependent on dialysis at discharge from the hospital.

**Table 6:** Follow-up of patients until discharge from the hospital

Follow-up	Death		Recovered		Acute kidney disease		Dialysis dependency at discharge (persistent AKI)	
	N	%	N	%	N	%	N	%
(Stage I)	5	12.5%	12	23.53%	0	0%	0	0%
(Stage II)	13	32.5%	22	43.14%	1	14.29%	2	33.33%
(Stage III)	22	55%	17	33.33%	2	66.6%	4	66.66%
Total	40	100%	51	100%	3	100%	6	

## Discussion

AKI occurs often in the geriatric population and is linked to increased mortality, morbidity, and medical expenses. Hospitalized individuals are three to six times more likely to die than the general population if they have AKI [8]. Despite improvements in medical care, more aggressive medical and surgical treatment, and associations with other comorbidities of aging, the incidence of AKI in the elderly has grown over time.

In our study, it was seen that 35 patients were more than 70 years of age, and 65 patients were 60-65 years of age. The majority of patients were males (70%), with 30% of patients being females, resulting in a male-to-female ratio of 2.33:1. About 60% of the patients in this study were admitted to medical ICUs, and 40% were admitted to the surgical ICU. In a study by Chertow et al., among geriatric patients, the mean age observed was 59.8 ± 17.1 years with 51% of the patients being females [13]. According to a study by Feest et al., patients aged more than 60 years are likely to develop community-acquired AKI three to eight times more frequently compared to the younger population [14]. According to a study by Prakash et al., the ratio of men to women was 2:1 for those who develop AKI after the age of 60 years (the average age was 72.5 years, with a range of 60-85 years) [15].

The most frequent etiology in this group was drug-induced, which accounted for 25% of cases, followed by age (21.67%), cardiac causes (13.33%), respiratory reasons (20%), tropical causes (15%), and pancreatitis (5%). The AKI in medical ICUs had a 41.67% mortality. Cellulitis (25%) was a significant etiology of AKI in surgical ICUs, followed by nonabdominal surgical procedures (12.5%) and intestinal obstruction (55%). The AKI in surgical ICUs had a 37.5% mortality rate. In a study by Kashinkunti et al., they found that there were 74% medical cases, 35% surgical cases, and 11% obstetrical cases in all age groups [16]. In a study by Turney et al. between 1980 and 1988, the median age of patients developing AKI increased from 41.25 years in 1950 to 60.5 years in 1980 [17]. According to Prakash et al., for geriatric patients with AKI, medical cases accounted for 80% of geriatric AKI, and 20% of patients had AKI due to surgical causes [15]. In a study by Mahajan et al., it was seen that drug-induced causes for AKI accounted for 11.5% of the total cases [18]. In the study by Prakash et al. on AKI in the elderly, it was seen that acute gastroenteritis was the main cause of AKI due to medical etiology [15]. In a study by Santacruz et al., the main cause of AKI in the elderly was sepsis, wherein respiratory failure-associated mortality was seen in two patients (7%) [19]. In a study by Queiroz et al. on AKI risk in geriatric patients with STEMI (ST-segment elevation myocardial infarction), 83 patients (20.4%) developed AKI, and mortality was seen in 11.8% [20].

The patient distribution was based on KDIGO criteria. There was no statistical significance for the difference in the proportion of mortality among the three stages of AKI (p=0.177). The most frequent AKI management was conservative (53%), followed by peritoneal dialysis (3%), and hemodialysis (44%). When a patient has one or more comorbid illnesses, their mortality is increased (p=0.027), which is statistically significant.

In the present study, the overall crude mortality rate was 40%. About 25% of patients were admitted to medical ICUs, while it was about 15% of patients in surgical ICUs. In a study by Pedersen et al., the mortality rate was 53.1%, while Martensson et al. demonstrated an overall mortality of 50% in geriatric patients with AKI [21,22]. 

In the present study, the commonest etiology of AKI was related to exposure to nephrotoxic drugs such as NSAIDs, aminoglycosides, diuretics, and ACE inhibitors. In a study, Yokota et al. observed that 21.9% of their patients had drug-induced kidney injury [23]. 

We observed that two-thirds of all patients with AKI (66.7%) required hemodialysis support. The mortality among patients undergoing peritoneal dialysis was higher (100%) compared to those undergoing hemodialysis (66.7%). A study by Kahindo et al. reported that among 131 patients with HD, 33 (25.19%) had died [24]. In a systemic review by Chionh et al., pooled mortality was 39.3% in the patients who were treated with peritoneal dialysis [25].

As the study was time-bound and included only the elderly population, only a small number of people were taken into the study. Moreover, there was no control group without AKI.

## Conclusions

This article attempts to sensitize readers about AKI in the geriatric population, which is a significant yet less-discussed issue in ICUs. According to our research, geriatric patients with AKI had a high overall mortality of 40%. The higher mortality was not associated with age as a singular factor. The higher mortality in the elderly was observed with a higher number of associated comorbidities and severity of AKI. We observed drug-induced AKI as a major contributor to hospital mortality, which should be a preventive ailment. AKI could be identified early in patients with multiple comorbidities to decrease the possibility of higher mortality in this subset of geriatric patients admitted to ICUs.
